# Identification of laccases involved in lignin polymerization and strategies to deregulate their expression in order to modify lignin content in Arabidopsis and poplar

**DOI:** 10.1186/1753-6561-5-S7-O39

**Published:** 2011-09-13

**Authors:** Jouanin Lise, Berthet Serge, Mazel Julien, Demont-Caulet Nathalie, Ayangma Brice, Le-Bris Philippe, Baratiny Davy, Leplé Jean Charles, Lapierre Catherine

**Affiliations:** 1 IJPB, UMR1318 INRA-AgroParisTech, 78026 Versailles, France; 2 AGPF, INRA, 45002 Ardon, France

## 

Lignins have a major impact on the agro-industrial uses of plants. Until now, most of the strategies considered for lignin reduction have targeted the monolignol pathway since the genes involved in these metabolic steps have been identified in many plants. Less is known about the other steps and in particular on lignin polymerization in the cell wall. While it is established that peroxidases are involved in the polymerization of lignin precursors, it is not yet clear whether laccases (EC 1.10.3.2) participate in constitutive lignification.

In order to address this issue, laccase genes (*AtLAC4* and *AtLAC17*) that are highly expressed in Arabidopsis stems were studied. *AtLAC17* was specifically expressed in the interfascicular fibers while *AtLAC4* was expressed in vascular bundles and interfascicular fibers. *Arabidopsis* T-DNA insertion mutants were selected and characterized. Two double mutants were obtained by crossing the *AtLAC17* (*lac17*) mutant with two *AtLAC4* mutants (*lac4-1* and *lac4-2*). The single and double mutants displayed normal growth, except the *lac4-2 lac17* mutant that sometimes had a semi-dwarf phenotype and collapsed vessels. While the single mutants had moderately reduced lignin levels, the stems of *lac4-1 lac17* and *lac4-2 lac17* had lignin content reduced by 20% and 40%, respectively. This lower lignin level improved their saccharification yield. Thioacidolysis revealed that disrupting *AtLAC17* mainly affected the deposition of G lignin units in the interfascicular fibers and that complementation of *lac17* with *AtLAC17* restored the normal lignin profile. This study provides evidence that both *AtLAC4* and *AtLAC17* contribute to the constitutive lignification of *Arabidopsis* stems and that *AtLAC17* is involved in the deposition of G lignin units in fibers, suggesting a role in early lignification (Berthet et al, in press).

The double mutants cannot be obtained for species that are propagated vegetatively such as poplar. In order to produce plants with lower laccase activity and reduced lignin content, we therefore used a miRNA strategy. The overexpression of two miRNA (miR397 and miR408) targeting several laccase genes was tested in different plants including Arabidopsis and poplar. These miRNAs were expressed constitutively under the control of the CaMV 35S promoter or of lignin-specific promoters such as *CAD* and *C4L* in transgenic Arabidopsis and poplar.

Results obtained using of this miRNA strategy in *Arabidopsi*s and preliminary results for poplar will be presented.

